# COVID-19 and palliative care capacity, African Region

**DOI:** 10.2471/BLT.20.285286

**Published:** 2021-08-01

**Authors:** Oladayo A Afolabi, Mary Abboah-Offei, Eve Namisango, Emeka Chukwusa, Adejoke O Oluyase, Emmanuel BK Luyirika, Richard Harding, Kennedy Nkhoma

**Affiliations:** a Florence Nightingale Faculty of Nursing, Midwifery and Palliative Care, Cicely Saunders Institute of Palliative Care, Policy and Rehabilitation, King’s College London, Bessemer Road, London, SE5 9PJ England.; b African Palliative Care Association, Kampala, Uganda.

Palliative care is included within the universal health coverage goal of the sustainable development goals as an essential health service and is considered a human right.^1^ The seventy-third World Health Assembly recommended palliative care as a core component within the coronavirus disease 2019 (COVID-19) response plans of Member States. However, the neglect of palliative care is a well established problem worldwide. Of the roughly 60 million people experiencing serious health-related suffering who would benefit from palliative care in a given year, over 80% live in developing countries where such care is scarce or non-existent.^2^ Mortality projections from the World Health Organization show an anticipated rapid increase in serious health-related suffering at the end of life in the coming decades.^3^ Delivering timely, appropriate and effective palliative care is a pressing challenge highlighted by the morbidity and mortality caused by COVID-19. 

COVID-19 patients and their families report multidimensional symptoms, ranging from those that are physical, to psychosocial, spiritual and existential distress from the threat to survival.^4^ In Africa, disease severity and case fatality rates are also influenced by population-specific factors, such as a young population, tuberculosis, cardiovascular diseases, metabolic conditions and immunodeficiency caused by human immunodeficiency virus (HIV) infection.^5^

In our recent review of African COVID-19 case management guidelines, we found few palliative care approaches and a focus on clinical management to the neglect of important psychosocial and spiritual stressors affecting morbidity and outcomes.^6^ Health systems’ allocation of resources to COVID-19 has severely weakened services for other serious conditions, such as cancer and HIV infection. Palliative care patients who contract COVID-19 are at risk of poorer outcomes, yet African palliative care providers have little COVID-19 preparedness and response capacity, as shown by a survey of African countries.^7^ Community outreach efforts to improve access to palliative care have been suspended. Furthermore, COVID-19 restrictions that hinder appropriate care delivery to the dying add to the distress of patients and their families, while social distancing and the ban on group gatherings disrupt the normal experience of grief and communal bereavement practices. In Africa, little has been done to develop the evidence base and adapt palliative care interventions to improve grief and bereavement outcomes.^8^ Research is required to address the evidence gaps and ensure that bereaved families receive appropriate support. 

The supply of medical products, including palliative medicines, has also been disrupted by the pandemic, even more so in countries still experiencing increased cases, including Kenya, South Africa and Uganda.^9^ Africa has limited capacity for manufacturing medicines such as morphine and receives less than 1% of the global supply of opioids. Before the pandemic, distributed opioids in Nigeria amounted to less than 1 mg per patient in need of palliative care per year – a mere 0.2% of the country’s annual need for morphine.^10^

Before the pandemic strained African health systems, only 5–11% of people requiring palliative care had access. The negative impact on services has heightened the risk of unnecessary suffering in patients with COVID-19 and other serious illnesses. Therefore, policy-makers in Africa must try to lessen the impact of the pandemic on palliative care services by integrating palliative care within the COVID-19 response. This integration involves training family caregivers and community health-care workers caring for COVID-19 patients and other seriously ill patients whose home care by specialist teams has been disrupted by pandemic restrictions, and ensuring ongoing funding for palliative care services.

African institutions must rapidly expand palliative care training to health-care professionals outside palliative care services and support the training with clear, comprehensive case management guidelines focused on palliative care. Service providers must also urgently collaborate with researchers in adapting palliative care delivery approaches and testing new technology-based service delivery models, such as mobile health applications.^11^ As the COVID-19 pandemic continues, African countries can seize the opportunity to improve health systems, mobilize resources and use innovation to strengthen palliative care.
